# Role of Radiotherapy in Metastatic Non-Small Cell Lung Cancer

**DOI:** 10.3389/fonc.2014.00229

**Published:** 2014-10-13

**Authors:** Sergio L. Faria

**Affiliations:** ^1^Department of Radiation Oncology, McGill University Health Centre, Montreal General Hospital, Montreal, QC, Canada

**Keywords:** palliative radiotherapy, metastatic NSCLC, stereotactic body radiosurgery, radiosensitizers

## Abstract

Radiotherapy has had important role in the palliation of NSCLC. Randomized trials tend to suggest that, in general, short regimens give similar palliation and toxicity compared to longer regimens. The benefit of combining chemotherapy to radiosensitize the palliative radiation treatment is an open question, but so far it has not been proved to be very useful in NSCLC. The addition of molecular targeted drugs to radiotherapy outside of approved regimens or clinical trials warrants careful consideration for every single case and probably should not be used as a routine management. Stereotactic radiosurgery (SRS) and stereotactic body radiation therapy (SBRT) are modern techniques being used each time more frequently in the treatment of single or oligometastases. In general, they offer good tumor control with little toxicity (with a more expensive cost) compared to the traditionally fractionated radiotherapy regimens.

## Introduction

Good palliative local treatment should be simple, fast, generally efficient, and not very expensive. Radiotherapy has largely been used to palliate NSCLC for all these reasons (1). A good example is the fact that one simple treatment of external beam radiation treatment (EBRT) may stop a hemoptysis. Multiple prospective randomized trials using different dose/fractionation schedules have shown that palliative radiotherapy can often alleviate thoracic and extra-thoracic symptoms in patients with locally advanced or metastatic NSCLC (1–3).

Indications for thoracic EBRT include, but are not limited to: hemoptysis, cough, chest pain, dyspnea, obstructive pneumonia, dysphagia related to esophageal compression, superior vena cava syndrome, hoarseness, or stridor. Symptoms caused by malignant pleural effusion, lymphangitic carcinomatosis, and multiple parenchymal diseases typically are not suitable for palliative thoracic EBRT.

Indications for extra-thoracic EBRT include, but are not limited to: brain, adrenal, bone, and liver metastases.

## Reviews

In 2009, a comprehensive review involving 14 randomized clinical trials, all related to different dose schedules to palliate the symptomatic primary lung cancer, was performed by the Cochrane Collaboration (4). In general, the results of those trials suggest that there are not significant differences among short compared to long radiotherapy regimens in terms of palliation, but higher-dose regimens were associated with mild increased acute toxicity, particularly esophagitis (Table 1). However, the studies are not homogeneous, the end points and assessments were different, and the reviewers did not make a clear conclusion on the ideal regimen of palliative radiation treatment. In fact, in clinical practice, depending on the institution, we have seen different doses and fractionations regimens being used for similar clinical situations. It is very well possible that, at least in part, remuneration directs practical management. The case for bone metastases is a good example. Despite a considerable body of evidence from randomized trials supporting the use of a single fraction of 8 Gy for radiation therapy, there is still considerable use of longer regimens such as 30 Gy in 10 fractions (5).

**Table 1:** **Randomized controlled trials assessing palliative lung radiotherapy fractionation [from Ref. (3)]**.

Study	Year	Radiotherapy schedules compared	Evaluable patients (*n*)	Survival^a^ by regimen (*P* = NS unless specified)	Symptom control by regimen (*P* = NS unless specified)
Simpson	1985	40 Gy/20 F daily continuous/4 weeks vs. 30 Gy/10 F/2 weeks vs. 40 Gy/10 F/4 weeks, split course	316	6.2 vs. 6.9 vs. 6.4 months	No difference
Teo	1988	45 Gy/18F/3.5 weeks vs. 31.2 Gy/4 F/4 weeks	273	20 vs. 20 weeks	Better with 45 Gy, *P* = 0.012
MRC	1991	30 Gy/10 F/2 weeks or 27 Gy/6 F/2 weeks or 17 Gy/2 F/8 days	369	177 vs. 179 days	No difference
MRC	1992	17 Gy/2 F/8 days vs. 10 Gy/1 fraction	235	100 vs. 122 days	No difference
Abratt	1995	35 Gy/10 F/2.5 weeks vs. 45 Gy/15F/3.75 weeks	84	8.5 vs. 8.5 months	No difference
MRC	1996	36 or 39 Gy/12 or 13 F/2.5 weeks vs. 17 Gy/2 F/8 days	509	1. 9 vs. 2.7 months, *P* = 0.03	No difference
Rees	1997	17 Gy/2 F/8 days vs. 22.5 Gy/5 F/5 days	216	23 vs. 18% (1 year)	No difference
Reinfuss	1999	50 Gy/25 F/5 weeks (conventional) vs. 40 Gy/10 F daily (split course with 4 weeks gap) vs. delayed radiotherapy (20 25 Gy/4 or 5 F when symptomatic).	240	18 vs. 6 vs. 0%, *P* < 0.05 (2 years)	No assessment of symptoms
Nestle	2000	32 Gy/16 F twice daily/10 days vs. 60 Gy/30 F/6 weeks	152	36 vs. 38% (1 year)	No difference
Bezjak	2002	20 Gy/5 F/1 weeks vs. 10 Gy/1 F	230	6 vs. 4.2 months, *P* = 0.03	Better for 20 Gy on Lung Cancer Symptom Scale, *P* = 0.009
Sundstrom	2004	17 Gy/2 F/8 days vs. 42 Gy/15 F/3 weeks vs. 50 Gy/25 F/5 weeks	407	6.8 vs. 7.0 vs. 8.2 months	No difference
Erridge	2005	30 Gy/10 F/2 weeks vs. 10 Gy/1 F	148	23 vs. 28 weeks	Better for 30-Gy arm, *P* = 0.05
Kramer	2005	30 Gy/10 F/2 weeks vs. 16 Gy/2 F/8 days	297	20 vs. 11%, *P* = 0.03 (1 year)	No difference
Senkus-Konefka	2005	20 Gy/5 F/l weeks vs. 16 Gy/2 F/8 days	100	5.3 vs. 8.0 months, *P* = 0.016	No difference

*F, fraction; Gy, gray; NS, non-significant*.

*^a^Survival given as median value or percentage at specific timepoint*.

## Typical Palliative Radiotherapy Dose and Fractionation in NSCLC

At the McGill University Health Centre (MUHC), we practice evidence-based medicine preferring short and simple treatments. Our typical palliative dose is 17 Gy in two fractions (1 week apart) after randomized trials concluded that this regimen is simple, well tolerated, and efficient compared to other regimens (6, 7).

## SVC Syndrome

In the past, SVC syndrome was considered a potentially life-threatening medical emergency requiring immediate radiotherapy as the quickest way to relieve the obstruction. Emergency radiotherapy is no longer considered necessary for most patients (8). Patients who present with stridor due to central airway obstruction or severe laryngeal edema represent a true medical emergency, and these patients require immediate treatment (stent placement and/or radiotherapy) to decrease the risk of sudden respiratory failure and death. Evidence-based guidelines for management of SVC syndrome are not available. Most of the malignancies causing SVC syndrome, including NSCLC, are radiation-sensitive, and symptomatic improvement is usually apparent within 72 h, associated with complete relief of symptoms of SVC obstruction in 63% of patients with NSCLC (9). Radiotherapy treatments are typically administered over a course of 1–2 weeks with larger fraction sizes of 3–8 Gy (e.g., 17 Gy in 2 fractions, 20 Gy in 5 fractions, and 30 Gy in 10 fractions), with the goal of achieving a more rapid response by using larger daily doses (10).

## Brachytherapy

In our Department at MUHC, we have the facilities to use high dose rate (HDR) endobronchial brachytherapy for palliation of hemoptysis or obstruction (we use doses between 6 and 10 Gy at 1 cm). Brachytherapy has been used sporadically. Comparing brachytherapy to EBRT is difficult. There is currently no randomized or meta-analysis based evidence to recommend endobronchial brachytherapy as the routine initial palliative management of endobronchial obstruction resulting from lung cancer (11).

The use of concurrent chemotherapy with palliative irradiation in lung cancer.

The question if some systemic treatment should be used as a radiosensitizer of palliative radiotherapy in NSCLC is open for discussion. The question is pertinent because several randomized studies have demonstrated that, when compared with best supportive care, chemotherapy not only significantly improves survival but also reduces symptoms and enhances quality of life in stage IV NSCLC. However, in palliative radiotherapy the total dose is usually not very high (to avoid risk of radiation induced toxicity). In general, in the group of metastatic NSCLC patients that need local radiotherapy palliation, the addition of chemotherapy may increase toxicity, cost, and may complicate the delivery of the whole treatment without significant improved palliation. At this time, it seems that there is no added benefit for the use of chemotherapy concurrently with radiation therapy in the palliation of thoracic symptoms in lung cancer patients (3, 4), but this is of course an open topic and indications may be discussed in a case by case basis. There is only one randomized clinical trial addressing this issue showing that the use of continuous-infusion fluorouracil showed only a discrete better response but with increased toxicity in palliation of NSCLC (12). However, fluorouracil is not an agent currently used in NSCLC, and the available data of palliative radiotherapy with the use of other agents commonly used today as systemic treatment in NSCLC such as platinum based chemotherapy, vinorelbine, or gemcitabine, are not very persuasive (13, 14).

## The Use of Target Agents with Palliative Radiotherapy

When used in combination with radiotherapy, molecularly targeted agents aim to increase the effect of the radiation on the tumor. Substantial preclinical data have accumulated to show that these agents can potentially enhance the tumor response to radiotherapy through a variety of mechanisms (15). They offer new but challenging possibilities for clinical practice. There is a growing number of publications and reviews on the topic of combination of radiotherapy and targeted therapies in many cancers, including NSCLC (16, 17). The addition of targeted agents to thoracic radiation so far has not improved outcomes in patients with locally advanced NSCLC (18, 19).

The combination of radiotherapy and molecular agents targeting vascular endothelial growth factor (VEGF) mediated angiogenesis may evolve synergistic effects leading to enhanced tumor cell killing on the one hand, but to enhanced normal tissue damage on the other hand (20). To date, there are only limited data on the efficacy and toxicity of anti-angiogenic agents given in combination with radiotherapy in lung cancer.

Given the strong preclinical rationale for combining EGFR inhibitors (Cetuximab, Panitumumab, Erlotinib, Gefitinib, Lapatinib, and Trastuzumab) with radiation, additional studies are crucial. Phase I/II data and lack of long-term experience suggest that physicians should consider combined modality approaches with caution, considering the possibility of uncommon but potentially severe toxicity (21).

With high-precision irradiation techniques (such as “stereotactic body radiation therapy”), the combination with targeted agents is feasible with apparent no increase in severe adverse events. Nevertheless, the addition of molecular targeted drugs to radiotherapy outside of approved regimens or clinical trials warrants careful consideration for every single case.

The problem of timing is particular to radiotherapy and molecularly targeted agent combination research. It cannot be assumed that giving the drug concurrently with radiation (as it happens with chemotherapy) is always the optimal treatment strategy. Indeed, drugs that cause cell cycle arrest or prolong cells in the radio-resistant phase of the cell cycle may jeopardize the radiation effect (17).

## Brain Metastases

When brain metastases occur in patients with NSCLC, there is often also active disease at the primary site or elsewhere in the body. In few cases, the brain is the only site with active disease (22).

There are many guidelines on the treatment of brain metastases showing that therapeutic intervention (radiotherapy or surgery) is associated with improved brain control (23).

Stereotactic radiosurgery (SRS) to the brain involves a single shot of high dose radiotherapy and can control very efficiently one to few metastases either close to the surface or deep in the brain (24). No randomized trials compared SRS with traditional surgical resection. The traditional whole brain radiotherapy (WBRT) (that covers the whole brain) treats the metastases and may also prevent the growth of new metastases, but may cause side effects such as memory loss. Recent Cochrane review shows that there is low quality evidence that adding upfront WBRT to surgery or to SRS decreases any intracranial disease progression at 1 year. There is also no clear evidence of an effect on overall and progression free survival (25).

Stereotactic radiosurgery has become increasingly important treatment technique in the management of brain metastases, but it is not available everywhere and it is more expensive than WBRT. An approach of SRS alone as initial treatment of brain metastases has allowed patients to delay or avoid WBRT and its associated side effects. “One of the most critical questions on this topic is how “benefit” is defined and from who’s perspective – patient, provider, payer, or society” (26). Whether the cost of SRS in multiple brain metastases versus just WBRT approach is justified has yet to be defined.

## Stereotactic Body Radiation Therapy

Stereotactic body radiation therapy or “stereotactic ablative radiotherapy” (SART) is a technique similar to SRS, but used in tumors outside the brain. It utilizes precisely targeted high dose radiation to the tumor while minimizing radiation to adjacent normal tissue. It has the luxury of using 4D CT scans to manage the pulmonary motion during treatments. This technique allows treatment of small to moderate sized tumors, in either a single or limited number of high daily dose fractions, with high chances of local control and little toxicity. SBRT has a role in treating selected patients with painful bone metastases or with oligometastases in lungs, liver, or other sites. In spine metastases, for example, non-randomized data show good results with this technique (27). Radiation Therapy Oncology Group (RTOG) study number 0631 is an open Phase II/III Study of Image-Guided SBRT for localized spine metastasis comparing one treatment of 16 Gy delivered with SBRT versus a single fraction of 8 Gy (28).

In patients with NSCLC and with oligometastases, there is a trend to treat them with SBRT, although there is no evidence-based data to show that SBRT is better than traditional palliative radiotherapy. Recently, a proposal submitted to RTOG was not approved because many participants would consider abusive not to offer SBRT in those cases. There is no prospective randomized trial to answer this question. The COMET study (stereotactic ablative radiotherapy for comprehensive treatment of oligometastatic tumors (SABR-COMET) is a randomized Phase II Trial (PIs: David Palma, and Suresh Senan) open in Europe and Canada, comparing patients with up to five metastatic lesions from any primary tumor site who can receive SABR. Eligible patients are randomized to either standard palliative radiotherapy versus SABR (with further chemotherapy at discretion of medical oncologist).

## Conflict of Interest Statement

The Guest Associate Editor Vera Hirsh declares that, despite being affiliated to the same institution as author Sergio Faria, the review process was handled objectively and no conflict of interest exists. The author declares that the research was conducted in the absence of any commercial or financial relationships that could be construed as a potential conflict of interest.
